# Prediction of Metabolizable Energy Concentrations of Herbage in the Qinghai–Tibetan Plateau Using Tibetan Sheep Digestibility Data

**DOI:** 10.3390/ani10030376

**Published:** 2020-02-26

**Authors:** Penghui Guo, Peng Gao, Fuhou Li, Shenghua Chang, Zhaofeng Wang, T Yan, Fujiang Hou

**Affiliations:** 1 State Key Laboratory of Grassland Agro-Ecosystems, Lanzhou University, Lanzhou 730030, China; cyguoph@163.com (P.G.); lifh17@lzu.edu.cn (F.L.); cychangsh@lzu.edu.cn (S.C.); wangzhf@lzu.edu.cn (Z.W.); 2 College of Pastoral Agriculture Science and Technology, Lanzhou University, Lanzhou 730030, China; 3 Key Laboratory of Grassland Livestock Industry Innovation, Ministry of Agriculture and Rural Affairs, Lanzhou University, Lanzhou 730030, China; 4 Agri-Food and Biosciences Institute, Hillsborough, County Down BT26 6DR, UK; t_yan82@yahoo.com

**Keywords:** alpine meadow, native herbage, metabolizable energy, prediction equations, sheep

## Abstract

**Simple Summary:**

Robust prediction of herbage nutritive value is critical to improve grazing efficiency and to maintain a sustainable environment in the Qinghai–Tibet Plateau. A range of prediction equations were developed in the present study using sheep digestibility data which can produce an accurate estimation of herbage nutritive value. The adaptation of the present equations is expected to benefit local farmers with higher economical return and to improve the fragile ecological systems the Qinghai–Tibet Plateau.

**Abstract:**

Due to its extremely harsh environment, including high altitude, hypoxia, long cold season, and strong ultraviolet radiation in the Qinghai–Tibet Plateau (QTP), herbage species and nutritional value of the pasture may differ considerably from elsewhere across the world. The aim of the present study was to develop biologically relevant equations for estimating the metabolizable energy (ME) value of fresh native herbages in the QTP using digestibility variables and chemical concentrations in the herbage offered to Tibetan sheep at the maintenance level. A total of 11 digestibility trials (6 sheep/trial) were performed in different grazing seasons from 2011 to 2016. The herbage was harvested daily in the morning and offered to sheep at the maintenance feeding level. Thirty-seven equations were developed for the prediction of herbage digestible energy (DE) and ME energy values. The mean prediction error for ME was the lowest when using herbage gross energy digestibility as a sole predictor. When using other digestibility variables (e.g., dry matter and organic matter) as primary predictors, addition of herbage nutrient concentration reduced the difference between predicted and actual values. When DE was used as the primary explanatory variable, mean prediction error was reduced with the addition of ash, nitrogen (N), diethyl ether extract (EE), neutral detergent fiber (NDF), and acid detergent fiber (ADF) concentrations. The internal validation of the present equations showed lower prediction errors when compared with those of existing equations for prediction of DE and ME concentrations in the herbage. Equations developed in the current study may thus allow for an improved and accurate prediction of metabolizable energy concentrations of herbage in practice, which is critical for the development of sustainable grazing systems in the QTP.

## 1. Introduction

The accurate prediction of herbage feed values is crucial for managing grassland sustainability and livestock production, especially in grazing areas with poor natural conditions and environment. This technique has been widely used in pasture-based systems in certain countries of the world to improve nutrient utilization efficiency of the herbage, animal production, and economic performance [1,2,3].

The Qinghai–Tibet Plateau (QTP) is the largest grassland area in the Eurasian continent and also the largest area of natural grasslands in China [4]. Grasslands, which cover about 30% of the total area, are the primary foundation of natural alpine meadow pastures in the QTP [5]. Approximately 50 million Tibetan sheep and 15 million yaks graze on these grasslands [6]. There are a range of concerns over the grazing systems. Perhaps, the primary concern is the huge variations in herbage production and quality between the grazing seasons (e.g., high herbage growth rate with good quality during the summer, and dead herbage with poor quality in the winter) [7,8]. The degradation of the natural grassland in the QTP caused by overgrazing and poor grazing management is another major problem [9]. Although many strategies have been undertaken in recent years with the aim of solving these problems, including grassland improvement, establishing sown pastures, and implementing reasonable grazing systems [10,11], the trend of the grassland degradation has intensified in recent decades, even causing serious economic and environmental problems for the local ecosystem and living standard of local farmers [12,13]. Therefore, more action and information are required to prevent grassland degradation in the QTP and improve the alpine rangeland productivity and herbage utilization efficiency. A key action is the development of an effective and rapid methodology to predict herbage nutritive value; in particular, developing tools to predict metabolizable energy (ME) in fresh grass may greatly improve profitability of pasture-based systems.

As one of the most important nutritive evaluation indices used in the world, feed metabolizable energy (ME) concentration is predicted using feed digestibility variables and chemical concentration [3,14,15]. Generally, the digestible energy (DE) or digestible organic matter in dry matter (DOMD), alone or in conjunction with the concentrations of herbage crude protein (CP), ash, acid detergent fiber (ADF), neutral detergent fiber (NDF), or diethyl ether extract (EE), are used to predict herbage ME concentration. However, limitations on the prediction of ME content of fresh grass raise concerns about the accuracy of ME predictions from grass nutrient contents and digestibility, such as the different proportions of digestible nutrients in DOMD which are not accounted for in the current UK feeding system [13]. In addition, the majority of works on the prediction of ME content of herbage were mainly performed in sheep and cattle production with diets based on conserved forages or single grass species [16,17,18].

In addition, Tibetan sheep graze on the natural alpine meadow, which has higher maintenance requirements compared with those used to develop the existing energy systems, and this may further contribute to potential errors when estimating grass ME contents in the QTP. 

It is therefore necessary to develop a quick and accurate evaluation methodology for prediction of fresh herbage ME concentrations based on the condition of the QTP. However, there is no such information available on the effects of seasonal change on herbage nutritive values and relationships between herbage ME concentration and nutrient digestibility and chemical composition in the QTP. Therefore, the aim of the present study is to develop prediction equations of herbage energy concentrations, particularly ME, from herbage digestibility parameters and nutrient concentrations for sheep fed native fresh-cut herbage, in an effort to quickly estimate grazing pasture ME contents of the alpine meadow and provide a scientific basis for the development of grazing animal diets, and to improve the management of native pastures for sustainable livestock productivity.

## 2. Materials and Methods

### 2.1. The Experimental Farm

The data used in the present study were collated from trials conducted from 2011 to 2016 in an experimental farm in Maqu County of Gansu Province, China, situated in eastern QTP (33°43′ N, 101°44′ E, 3600 m a.s.l.). The mean annual precipitation was about 600 mm in the past 50 years, and average precipitation levels during the growing (June–mid-September) and cold seasons (November–March) were 345 mm and 99 mm, respectively. The mean annual temperatures for the growing and cold seasons were 11.28 °C and 0.28 °C, respectively. The experiment farm has an average annual sunshine of 2580 h and more than 270 frost days per year [4]. The soil is dark black in color as a result of transformation of plant material to humus in the cold weather [13]. The plant community at the site is mainly dominated by the alpine meadow of Cyperaceae (especially Kobresia gramophone C.B. Clarke) with some Poaceae (Agrostis masquerade Hack. Ex Honda, Festuca errata Keng ex. E.B. Alexeev, and Poa annum L.). Various dicots are also presented including members of the Ranunculaceae, Polygonaceae, Saxifragaceae, Asteraceae, Scrophulariaceae, Gentianaceae, and Fabaceae [4,6].

### 2.2. Animal Handling and Housing

The animal sampling procedure strictly followed the rules and regulations of the Biological Studies Animal Care and Use Committee of Gansu Province, China (2005–2012) and the experimental field management protocols (file No: 2010-1 and 2010-2), which were approved by Lanzhou University. 

Before the commencement of the experiment, sheep were treated against internal parasites with albendazole and external parasites were eradicated. The sheep were housed in individual metabolic cages for 3 weeks, with 14 days for diet adaptation and 7 days for collection of feed intake and feces and urine output. Animals had free access to water throughout the adaptation and measurement periods. In each trial period, the fresh forage of alpine meadow was harvested daily in the morning from experimental fields and chopped into 1–2 cm length before feeding.

### 2.3. Experimental Design

The present data were collated from 11 digestibility trials carried out in June of 2012 and 2013; August of 2011, 2012, 2013, 2015, and 2016; and December of 2011, 2012, 2015, and 2016, respectively. A total of 66 male Tibetan sheep, two years of age and 29.8 ± 8.06 kg live weight, was used, which were selected from the experimental farm with 6 sheep in each trial. For a quick assessment of amounts required to meet maintenance energy requirements, fresh herbage DM concentration was estimated by microwaving at full power for 3–5 min and herbage ME was predicted using the methods of Stergiadis and colleagues [3]. The maintenance ME requirements of the Tibet sheep were calculated using the method of the Agriculture and Food Research Council (AFRC) [19].

### 2.4. Sampling

Sheep were weighed at the beginning and end of trials before the morning feeding. Daily feed intake, residual, feces, and urine were recorded, and samples were collected at the final 6 days and stored at −20 °C for further analysis. Fresh native herbage, offered to the sheep each day, was thoroughly mixed before sampling and each sample was divided into 2 subsamples for analysis of dry matter (DM) and herbage nutrient concentrations. Fresh feces and urine outputs were collected daily. Urine output was collected using a urine collector, which was fixed using cannas belts around the prepuce of the sheep, with urine flowing through a tube to a marked collector containing 10% HCl solution. The feed, feces, and residue samples were dried at 65 °C for 24 h and ground to pass a 1 mm screen and then preserved in self-sealed plastic bags for analysis of gross energy (GE), ash, nitrogen (N), EE, ADF, and NDF concentrations.

### 2.5. Chemical Analysis

The ground samples were dried in a forced-air oven at 135 °C for 2 h to calculate DM of forage and feces [20]. The ash concentration of herbage, weighed from a portion of the dried sample, was burned in a muffle furnace at 550 °C for 4 h until all carbon was removed, and then reweighed and calculated. The concentration of GE in herbage was determined in an isoperibol bomb calorimeter (6400, PARR Inc., Moline, IL, USA). The total N concentration in feed and feces was determined using the method of Kjeldahl and CP concentration was calculated using N concentration × 6.25. The NDF and ADF concentrations were analyzed sequentially using an ANKOM 2000 Fiber Analyzer (ANKOM Technology, Fairport, NY, USA) following the protocol described by Goering and Van Soest et al. [21,22]. Neutral detergent fiber and ADF were determined according to Goering [21] and Van Soest et al. [22], respectively. The EE was analyzed using an ANKOM XT15 Extractor (ANKOM Technology). Urine N concentration was determined as described above. Urine GE concentration was measured, using a 10 mL freeze-dried urine sample in a self-sealing polyethylene bag of known weight and energy concentration [23]. The ME concentration was calculated using digestible energy (DE) intake, urine energy output, and methane energy emission. Methane emissions were estimated using the data measured by the sulfur hexafluoride tracer technique in the first year of the present study [24].

### 2.6. Calculations

Linear regression relationships were developed with DE and ME concentrations and ratios of DE/GE and ME/GE as the responses; the fixed terms were: (1) digestibility of N (ND), GE (GED), NDF (NDFD), ADF (ADFD), DM (DMD), OM (OMD) and digestible organic matter in DM (DOMD); (2) herbage concentrations of N, GE, EE, NDF, ADF, and ash; (3) DE concentration for prediction of ME concentration; and (4) total digestible CP (tdCP) and total digestible NDF (tdNDF). These equations were developed either in univariate (Equation (1)) or multivariate (Equation (2)) linear models.

*Y* = *a* + *bx* (1)

*Y* = *a* + *b_1_x_1_* + *b_2_x_2_* + *b_3_x_3_* + … + *b_n_x_n_*. (2)

Candidate nested models of random variation, with the same fixed-effect model, were compared using the deviance. The change in deviance between the nested random models was assessed using χ^2^ with the *df* given by the difference in *df* of the two models. The significance of the fixed terms was assessed using the Wald statistic. The squared correlation of the response and the fitted values (R^2^) to represent the amount of variability explained was also derived.

The internal evaluation, described by Stergiadis et al. [3] and Yan [25], was performed to validate all prediction equations developed in the present study. The whole dataset (*n* = 66) was divided into two sub-datasets of *n* = 44 (two-thirds of the total data) and *n* = 22 (one-third of the total data). The former dataset was used to develop similar equations to those using the whole dataset, while the latter was used to evaluate these new equations. The validation was undertaken using the mean square prediction error (MSPE) technique (Equation (3)).

(3) $$\mathrm{MSPE}=1/n\sum {\left(P-A\right)}^{2}$$

where P and A are the predicted and actual values, respectively, and n is the number of pair of values of P and A compared. Mean prediction error (MPE) was used to describe the prediction accuracy (Equation (4)):

(4) $$\mathrm{MPE}=\sqrt{\mathrm{MSPE}}\;/(\sum A/n)$$

The same one-third of the present dataset was also used to validate the equations of Stergiadis et al. [3], Terry et al. [15], Givens et al. [16], AFRC [19], Zhang et al. [26], Zhao et al. [27], and the National Research Council (NRC). [28], for the prediction of ME concentrations, using DE or digestibility and herbage chemical concentrations parameters.

### 2.7. Statistical Analysis

Data were analyzed using the GenStat statistical package (2013). The residual diagnostics were assessed using normality plots. Prediction equations were developed using residual maximum likelihood (REML) [29], with random effects of sheep, season and year, removed.

## 3. Results

### 3.1. Herbage Chemical Composition and Digestibility Parameters

The mean, standard deviation (SD), minimum and maximum values for herbage chemical composition, nutrient digestibility, and energy concentrations are presented in Table 1. There were large variations in herbage chemical composition between the maximum and minimum values. The maximum concentrations of herbage DM, EE, and N were two to three times their minimum values; and ash, NDF, and ADF were approximately 1.5, 1.7, and 1.4 folds, respectively. However, GE concentrations were relatively consistent, ranging from 17.3 to 18.7 MJ/kg DM. Consequently, there were large variations in herbage digestibility variables and energy concentrations. For example, N and GE digestibility had maximum values more than 2.5 and 3.5 times the minimum data, respectively. The maximum value for DE or ME concentrations was 1.9 or 2.1 times the minimum value.

### 3.2. Development of Prediction Equation for DE and ME Concentrations 

The equations for prediction of DE and ME concentrations and ME/GE and DE/GE ratios using digestibility variables as the explanatory variables are presented in Table 2. All relationships were positive and significant. The organic matter digestibility (OMD) is the best explanatory variable for prediction of DE concentration (R^2^ = 0.886), while the highest R^2^ value (0.961) was obtained using GED to predict the ME concentration. However, for prediction of ME/GE, the R^2^ value relating to DOMD was higher than GED (0.868 vs. 847). For prediction of DE/GE, the R^2^ values were similar when using OMD, DMD, GED, and DOMD as predictors, although it was marginally higher with OMD (R^2^ = 0.832). 

The multiple linear equations for prediction of DE and ME concentrations and ME/GE and DE/GE ratios using digestibility data and nutrient concentrations are presented in Table 3. When chemical composition variables of herbage were added to support DMD, OMD, DOMD, or GED, R^2^ values for prediction of DE and ME concentrations and ME/GE and DE/GE ratios were increased, with all values higher than 0.8, except for prediction of ME/GE using DMD as the primary predictor (R^2^ = 0.794).

The equations for prediction of ME concentration using DE concentration as the primary predictor are presented in Table 4. The DE alone is a very good predictor for ME concentration (R^2^ = 0.905), adding N, EE, ash, and ADF only marginally increased the R^2^ value to 0.917, although all had a significant effect on the relationship between ME and DE concentrations.

The prediction equations for DE and ME concentrations using tdCP and tdNDF as primary predictors are presented in Table 5. The R^2^ values were similar for prediction of DE (0.797) and ME (0.798) concentrations using tdCP and tdNDF. Adding EE or ash concentration as a supporting predictor for DE or ME concentration increased the R^2^ value to 0.815 or 0.817, respectively.

### 3.3. Internal Validation to Assess the Prediction Equations

Thirty-nine new predictions, which were developed from two-thirds of the whole data using similar random and fixed factors to those developed using the whole data, are presented in Table 6 (equations A–AM). These new equations were then validated using the remaining one-third of the whole data. The validation results are presented in Table 7. The predicted values are very close to the actual data for all energy concentration and ratio parameters. The MPE were lower for the predictions of DE (average = 0.013), DE:GE (average = 0.015), and ME:GE (average = 0.019) than the ME (average = 0.033). The results of average mean difference values and mean standard errors between predicted and actual values are similar to those of MPE, and the R^2^ values between predicted and actual values from low to high are in the order for prediction of ME:GE, DE, ME, and DE:GE. 

Some published equations [3,15,16,19,26,27,28,30], for prediction of ME concentrations from DE or nutrient digestibility and concentrations, were also validated using the same one-third of the present data. The results are presented in Table 8 (equations AN–AZ). The MPE values for these published equations are higher than the equations developed in the present study. The result, predicted by Zhang et al. [26] and Zhao et al. [27], showed the lower MPE when using DE as the sole predictor compared with that of DOMD, DMD, or CP.

**Figure 1:**
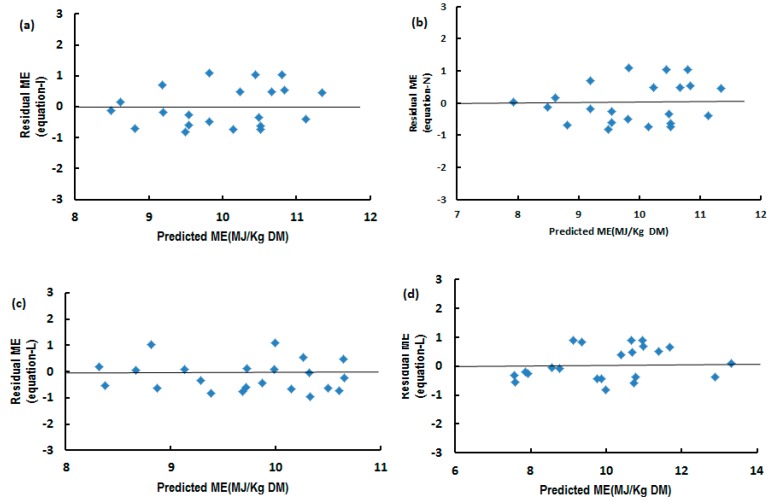
The relationship between residual ME concentration (predicted–actual) and predicted ME concentration from equations of I (**a**), N (**b**), L (**c**) and L (**d**) using one-thirds of the present data.

The Lin’s concordance correlation coefficient (R_c_) was used for further verification of differences between predicted and actual values using equations developed in the present study and by previous authors [3,15,16,19,26,27,28,30]. The results are presented in Table 7 and Table 8, respectively. The residual differences between predicted and actual ME values for some present equations are presented in Figure 1. They all recorded a high agreement in R_c_ between predicted and actual ME values. The scatter of differences between predicted and actual values was smaller for the equations developed by using digestibility parameters and nutrient concentrations as predictors (Figure 1). 

## 4. Discussion

This study has some unique aspects when compared to previous studies on the development of prediction equations for herbage ME concentration [31,32,33,34,35,36]. This is the first study to develop prediction equations for ME concentrations of native herbages in the QTP using digestibility data measured with Tibetan sheep fed fresh herbage of native alpine meadow in the whole typical grazing season. These prediction equations may be used for a range of grazing native alpine meadow pasture, because they were developed with sheep fed fresh-cut native herbage of highly diverse ingredients and may be used accordingly for different types of available analyses. The majority of previous prediction equations published elsewhere was carried out with sheep offered a single cultivated herbage [3,17]. The QTP is the largest and highest alpine regions and has the most fragile environment in the world. The herbage feeding values in the QTP vary considerably with seasonal change (e.g., herbage DM concentration varied from 323 to 809 g/kg in this study). The development of an evaluation tool to predict herbage feeding values could certainly help establish sustainable grazing systems to protect the QTP’s fragile environment.

This study showed that DE and ME concentrations increased with increasing DMD, OMD, DOMD, or GED, which is similar to those reported with fresh herbage [3] or frozen grass [2]. The best single predictor for ME concentration in the current study is GED, because it takes account for the majority of energy losses [17]. However, using GED as the predictor for ME concentration is not convenient when compared with DMD, especially under the grazing-pasture system in the QTP. Among the other three digestibility variables, the present study found that DOMD was a more accurate predictor for DE and ME concentrations with smaller MPE values and higher R^2^ data, when compared with equations using DMD or OMD as a sole predictor. This may be due to the fact that the measurement of DOMD takes account for the effect of ash concentration which reflects the real digestion and utilization of OM in herbage by the animal. Moreover, MPE value is also relatively small when using either DMD or OMD as the single predictor, which could be used for forecasting ME concentration of fresh herbage if GED or DOMD is unavailable.

Adding chemical variables to support digestibility factors for prediction of herbage DE and ME concentrations significantly improved prediction accuracy [37]. Using these chemical variables can also predict ME concentrations which are closer to the actual values when compared to those predicted using nutrient digestibility as the primary predictor [18]. The present study found a similar result in that adding herbage N, GE, NDF, and ADF concentrations to equations using digestibility variables as sole predictors significantly reduced MPE values and increased R^2^ data. The improvement of the prediction accuracy by adding chemical composition may be because these chemical composition data minimize their effects on chemical variations in the digestible nutrient concentrations per kg DM intake, because digestible fat, protein, and carbohydrates (39.5, 24.5, and 17.0–19.0 MJ/kg, respectively) contain different levels of energy [19].

The prediction of ME concentration in the current study had relatively low MPE and high R^2^ values when using DE concentration alone or DE concentration combined with herbage nutrient concentrations. This result is similar to those reported previously [3]. In the present study, although DE concentration was a relatively accurate predictor for ME concentration, the present internal validation found that adding nutrient concentrations including herbage N, ADF, EE, and ash, improved the prediction accuracy with a lower MPE in the relationship between predicted and actual values. This result is in agreement with previous work [3,15,18]. In contrast to the complicated measurement of herbage ME concentration, calculation of DE values does not require measurements of urine and methane energy emissions and is widely used to develop the equations for practical prediction of ME (e.g., Stergiadis et al. [37]).

Herbage digestible nutrient concentrations are recommended as explanatory variables to predict herbage DE and ME concentrations. These prediction models [3,15,16,17,19,26,27,28,29,30] were developed with animals fed either ad lib or at the maintenance level on single species sown grass, so possibly having some limitations for use with grazing systems in the native grassland. As discussed previously, the native herbage in the QTP has some special aspects, due to the unique climate and environmental condition in this region. The present study used tdCP and tdNDF as primary predictors, in conjunction with herbage EE and ash, for development of prediction equations for herbage DE and ME concentrations. However, these relationships had a relatively lower R^2^ value and higher MPE data in the internal validation when compared to those developed using digestibility data and nutrient concentration in the present study. 

The valuation using the same one-third of data in the present study also found high MPE values for the previously published prediction equations using total digestible nutrient concentrations [3,15,16,17,26,27,28]. However, when digestibility parameters and herbage nutrient concentrations were used to predict ME, the mean residual (predicted minus actual) was smaller for the present equations than those developed in previous publications [3,15,16,17], especially for the recommended equation from the NRC [28] using DE as sole predictor. These results indicate that using previous equations to predict herbage ME concentration in the QTP might cause errors. Although using DE as the sole predictor showed relatively low MPE in this study, the inclusion of N, EE, ash, and ADF contents of herbage as predictors may be recommended for further improvement of prediction accuracy in practice. Moreover, we observed herbage N content as a significant predictor of ME when using DE as the primary predictor; the addition of N and EE decreased MPE by 50% when compared with the equation using DE as the sole predictor. Therefore, using herbage N content as an additional predictor may be recommended when other predictors are not available in practice. This may be used for animal grazing in native grass and pasture-based systems as described in alpine meadow of the QTP. 

## 5. Conclusions

The present study confirms that the use of a combination of chemical composition of herbage and nutrient digestibility parameters could improve the accuracy of the prediction of energy concentrations in fresh herbage. Meanwhile, the developed equations in this study using GED or OMD could also be reliable alternatives to predict ME for their low prediction errors, and recommended instead of equations using DOMD. When ME was predicted by using DE as the sole predictor, compared with existing equations, the updated equations formulated may reduce prediction error for grazing sheep in alpine meadow; and also may be used as a relatively reliable technique to improve accuracy on a commercial scale because of their lower prediction errors when ME is predicted by using DE connection with herbage nutrient contents (N and EE; or EE, ADF, and ash), compared with existing equations used in the US and the UK feeding systems. The present equations may be used for animal grazing in native grass and pasture-based systems as described in alpine meadow of the QTP.

## Figures and Tables

**Table 1:** Herbage nutrient concentration and digestibility and energy utilization data used in the present study.

Parameter Assessed	Mean	SD	Min ^1^	Max ^2^
Herbage Nutrient Concentration (g/kg DM)				
DM (g/kg fresh)	566	272.3	323	809
N	64	36.4	35	124
Neutral detergent fiber	643	105.4	499	867
Acid detergent fiber	329	46.7	281	401
Diethyl ether extract	33	8.9	17	50
Ash	62	8.2	51	75
Gross energy (MJ/kg DM)	17.55	0.45	17.03	18.25
Nutrient digestibility (g/g)				
Dry matter	0.655	0.088	0.476	0.831
GE (MJ/MJ)	0.652	0.104	0.461	0.829
Organic matter	0.681	0.071	0.550	0.773
N	0.557	0.202	0.327	0.793
Neutral detergent fiber	0.702	0.064	0.542	0.811
Acid detergent fiber	0.648	0.056	0.519	0.773
DOMD	0.622	0.076	0.499	0.731
Energy Concentration and Utilization				
DE (MJ/kg DM)	11.53	2.15	8.03	15.21
ME (MJ/kg DM)	9.89	1.84	6.33	13.36
DE/GE (MJ/MJ)	0.648	0.092	0.461	0.839
ME/GE (MJ/MJ)	0.556	0.093	0.363	0.737

N, nitrogen; DE, digestible energy content; ME, metabolizable energy content; GE, gross energy content; DOMD, digestible organic matter in dry matter. ^1^ Min, minimum value observed; ^2^ Max, maximum value observed.

**Table 2:** Linear prediction equations for herbage energy concentrations (MJ/kg DM) and ratios using nutrient digestibility parameters.

Parameters	Equations	R^2^	MPE ^1^	Eq. No
DE =	−0.527_(0.827)_ + 18.552_(1.255)_DMD	0.806	0.031	1a
DE =	−0.614_(1.209)_ + 18.839_(1.918)_DOMD	0.850	0.024	1b
DE =	−2.535_(1.331)_ + 20.689_(1.941)_OMD	0.886	0.021	1c
ME=	−2.538_(1.12)_ + 19.92_(1.778)_DOMD	0.871	0.044	2a
ME=	−2.279_(0.84)_ + 18.692_(1.275)_DMD	0.805	0.042	2b
ME=	−4.803_(1.256)_ + 21.6_(1.832)_OMD	0.878	0.038	2c
ME=	−2.07_(0.339)_ + 18.146_(0.507)_GED	0.961	0.033	2d
ME/GE =	0.055_(0.053)_ + 0.881_(0.08)_DMD	0.699	0.041	3a
ME/GE =	−0.16_(0.052)_ + 1.16_(0.075)_OMD	0.823	0.037	3b
ME/GE =	0.068_(0.038)_ + 1.116_(0.06)_DOMD	0.868	0.046	3c
ME/GE =	0.061_(0.034)_ + 0.862_(0.051)_GED	0.847	0.032	3d
DE/GE =	−0.004_(0.044)_ + 1.019_(0.066)_DMD	0.819	0.034	4a
DE/GE =	−0.19_(0.053)_ + 1.248_(0.078)_OMD	0.832	0.023	4b
DE/GE =	0.063_(0.048)_ + 1.158_(0.076)_DOMD	0.819	0.035	4c
DE/GE =	0.097_(0.047)_ + 1.186_(0.073)_GED	0.828	0.023	4d

Data in subscript parentheses are standard error of means. DE, digestible energy content (MJ/kg DM); ME, metabolizable energy content (MJ/kg DM); GE, gross energy content (MJ/kg DM); DMD, DM digestibility (kg/kg), OMD, OM digestibility (kg/kg), DOMD, digestible OM in DM (kg/kg); GED, GE digestibility (MJ/MJ). ^1^ Mean prediction error (MPE) derived from an internal validation with new equations, listed in Table 6, which were developed from two-thirds of the whole dataset and by using the exact model presented in the current table; the new equations were validated against the remaining one-third of the whole dataset.

**Table 3:** Multivariate linear prediction of herbage energy concentrations (MJ/kg DM) and ratios using nutrient digestibility and chemical composition parameters.

Parameters	Equations	R^2^	MPE ^1^	Eq. No
DE=	−17.236_(5.379)_+15.057_(2.477)_DMD+20.716_(7.674)_N+3.194_(4.904)_ADF+0.909_(0.26)_GE	0.853	0.018	1d
DE=	−15.244_(4.292)_+9.74_(3.005)_OMD+28.34_(8.941)_N+3.789_(1.512)_NDF+0.862_(0.256)_GE	0.875	0.022	1e
DE=	−4.546_(2.243)_+13.431_(2.906)_DOMD+34.783_(6.328)_N+7.521_(1.597)_NDF	0.869	0.032	1f
ME=	−15.57_(1.435)_+18.111_(0.549)_GED+2.864_(2.351)_N+4.138_(1.31)_ADF+1.951_(0.368)_NDF+0.598_(0.069)_GE	0.991	0.042	2e
ME=	−15.999_(3.909)_+13.743_(2.509)_DMD+5.472_(5.952)_N+0.926_(0.242)_GE	0.853	0.039	2f
ME=	−12.64_(4.048)_+14.334_(2.834)_OMD+15.983_(6.676)_N+3.156_(1.426)_NDF+1.092_(0.241)_GE	0.848	0.038	2g
ME=	−13.15_(4.073)_+8.289_(2.519)_DOMD+13.034_(5.375)_N+63.697_(15.664)_EE+0.822(0.243)GE	0.861	0.040	2h
ME/GE=	−0.267_(0.163)_+0.786_(0.105)_DOMD+0.701_(0.231)_N+0.02_(0.01)_GE	0.871	0.042	3e
ME/GE=	0.156_(0.044)_+0.623_(0.091)_GED+0.805_(0.261)_N	0.794	0.034	3f
ME/GE=	−0.172_(0.232)_+0.244_(0.149)_DMD+1.598_(0.353)_N+0.028_(0.014)_GE	0.866	0.038	3g
ME/GE=	−0.464_(0.216)_+0.772_(0.152)_OMD+0.789_(0.285)_N−0.446_(0.856)_EE+0.029_(0.012)_GE	0.897	0.043	3h
DE/GE=	0.351_(0.078)_+0.562_(0.111)_DMD−0.306_(0.061)_NDF+4.123_(1.054)_EE	0.842	0.037	4e
DE/GE=	−0.873_(0.184)_+1.027_(0.087)_OMD+1.719_(0.688)_N+0.041_(0.011)_GE	0.876	0.022	4f
DE/GE=	0.079_(0.054)_+0.651_(0.116)_DOMD+0.814_(0.25)_N+3.238_(0.675)_EE	0.894	0.024	4g
DE/GE=	0.079_(0.054)_+0.651_(0.116)_GED+0.814_(0.25)_N+3.238_(0.675)_EE	0.878	0.021	4h

Data in subscript parentheses are SE values. ADF, acid detergent fiber (kg/kg DM); NDF, neutral detergent fiber (kg/kg DM); N, nitrogen (kg/kg DM); EE, diethyl ether extract (kg/kg DM); DE, digestible energy content (MJ/kg DM); ME, metabolizable energy content (MJ/kg DM); GE, gross energy content (MJ/kg DM); DMD, DM digestibility (kg/kg); OMD, OM digestibility (kg/kg); DOMD, digestible OM in DM (kg/kg); GED, GE digestibility (MJ/MJ). ^1^ MPE derived from an internal validation with new equations, listed in Table 6, which were developed from two-thirds of the whole dataset and by using the exact model presented in the current table; the new equations were validated against the remaining one-third of the whole dataset.

**Table 4:** Prediction equations for herbage ME concentration using DE concentration and chemical composition parameters.

Parameters	Equations for the Prediction of ME	R^2^	MPE ^1^	Eq. No
ME=	−1.38_(0.502)_ +0.964_(0.043)_DE	0.905	0.043	2i
ME=	−0.957_(0.659)_+0.937_(0.078)_DE+1.757_(4.095)_N	0.906	0.029	2j
ME=	−0.67_(0.653)_+0.814_(0.096)_DE+3.555_(4.064)_N+28.425_(13.741)_EE	0.913	0.022	2k
ME=	0.568_(1.047)_+0.785_(0.081)_DE−1.624_(0.936)_ADF+40.838_(15.718)_EE−12.531_(10.124)_Ash	0.913	0.031	2l
ME=	−1.403_(1.821)_+0.893_(0.062)_DE−14.506_(12.345)_Ash−5.685_(2.829)_ADF	0.917	0.028	2m

DE, digestible energy content (MJ/kg DM); ME, metabolizable energy content (MJ/kg DM); ADF, acid detergent fiber (kg/kg DM); N, nitrogen (kg/kg DM); EE, diethyl ether extract (kg/kg DM). Data in subscript parentheses are SE values. ^1^ MPE derived from an internal validation with new equations, listed in Table 6, which were developed from two-thirds of the whole dataset and by using the exact model presented in the current table; the new equations were validated against the remaining one-third of the whole dataset.

**Table 5:** Prediction equations for herbage DE and ME concentrations using total digestible nutrient concentration and chemical composition parameters.

Parameters	Equations for the Prediction of DE and ME	R^2^	MPE ^1^	Eq. No
DE =	5.706_(0.906)_ +0.053_(0.004)_ tdCP+0.007_(0.002)_ tdNDF	0.797	0.056	1g
DE =	6.397_(0.932)_+0.043_(0.006)_ tdCP+0.002_(0.003)_ tdNDF+55.374_(25.673)_ EE	0.815	0.045	1h
ME =	3.581_(0.916)_+0.054_(0.004)_ tdCP+0.008_(0.002)_ tdNDF	0.798	0.077	2n
ME =	4.314_(0.938)_+0.042_(0.006)_tdCP+0.003_(0.003)_ tdNDF+58.789_(25.837)_ Ash	0.817	0.062	2o

DE, digestible energy content (MJ/kg DM); ME, metabolizable energy content (MJ/kg DM); EE, diethyl ether extract (kg/kg DM); CP, crude protein (kg/kg DM); NDF, neutral detergent fiber (kg/kg DM); tdCP, total digestible CP content (g/100 g DM); tdNDF, total digestible NDF content (g/100 g DM). Data in subscript parentheses are SE values. ^1^ MPE derived from an internal validation with new equations, listed in Table 6, which were developed from two-thirds of the whole dataset and by using the exact model presented in the current table; the new equations were validated against the remaining one-third of the whole dataset.

**Table 6:** Internal validation: prediction equations for DE and ME concentrations and ratios developed using two-thirds of the whole dataset (*n* = 44).

Original Eq. ^1^	New Eq. ^2^	Parameters	Equations	R^2^	MPE ^3^
1a	A	DE =	−0.81_(1.596)_^C^+18.928_(1.596)_DMD	0.825	0.031
1b	B	DE =	−2.877_(1.649)_+21.073_(2.41)_OMD	0.872	0.024
1c	C	DE =	−0.844_(1.49)_+19.673_(2.363)_DOMD	0.891	0.021
1d	D	DE =	−24.014_(8.749)_+15.133_(3.095)_DMD+27.356_(9.718)_N+3.107_(6.006)_ADF+1.3_(0.337)_GE	0.875	0.018
1e	E	DE =	−20.382_(5.405)_+8.572_(3.757)_OMD+28.302_(8.959)_N+2.749_(1.9)_NDF+1.235_(0.331)_GE	0.878	0.022
1f	F	DE =	−4.71_(2.912)_+13.898_(3.819)_DOMD+35.073_(8.597)_N+7.281_(2.099)_NDF	0.859	0.032
1g	G	DE =	5.282_(1.19)_+0.056_(0.005)_tdCP+0.007_(0.002)_tdNDF	0.858	0.056
1h	H	DE =	6.342_(1.206)_+0.042_(0.008)_tdCP+0.001_(0.004)_tdNDF+75.792_(32.468)_EE	0.836	0.045
2a	I	ME =	−2.219_(1.035)_+18.685_(1.581)_DMD	0.854	0.044
2b	J	ME =	−4.742_(1.5)_+21.511_(2.191)_OMD	0.899	0.042
2c	K	ME =	−2.918_(1.295)_+20.484_(2.053)_DOMD	0.897	0.038
2d	L	ME =	−2.14_(0.41)_+18.212_(0.614)_GED	0.963	0.033
2e	M	ME =	−15.32_(5.095)_+10.427_(3.205)_DMD+15.679_(7.663)_N+1.055_(0.322)_GE	0.862	0.042
2f	N	ME =	−20.38_(5.405)_+8.572_(3.757)_OMD+28.302_(8.959)_N+2.749_(1.9)_NDF+1.235_(0.311)_GE	0.894	0.039
2g	O	ME =	−11.54_(5.288)_+2.979_(3.16)_DOMD+26.118_(6.836)_N+67.1_(19.978)_EE+0.942(0.317)GE	0.897	0.038
2h	P	ME=	−15.56_(5.381)_+14.95_(2.31)_GED+23.02_(8.669)_N+9.328_(4.557)_ADF+3.805_(1.284)_NDF+0.552_(0.286)_GE	0.935	0.040
2i	Q	ME =	−0.986_(0.544)_+0.949_(0.047)_DE	0.924	0.043
2j	R	ME =	−0.557_(0.703)_+0.881_(0.085)_DE+4.557_(4.714)_N	0.926	0.029
2k	S	ME =	−0.356_(0.722)_+0.795_(0.114)_DE+6.143_(4.903)_N+19.075_(16.972)_EE	0.929	0.022
2l 2m	T U	ME =	4.491_(1.955)_+0.867_(0.063)_DE−30.28_(13.157)_Ash−8.315_(3.076)_ADF	0.942	0.031
		ME =	1.324_(1.248)_+0.766_(0.098)_DE−2.316_(1.152)_NDF+37.532_(19.898)_EE	0.933	0.028
2n	V	ME =	3.717_(1.128)_+0.056_(0.005)_tdCP+0.008_(0.002)_tdNDF	0.823	0.077
2o	W	ME =	5.556_(1.457)_+0.062_(0.005)_tdCP+0.008_(0.002)_tdNDF−38.612_(20.369)_Ash	0.841	0.062
3a	X	ME/GE =	−0.01_(0.051)_+0.945_(0.078)_DMD	0.813	0.041
3b	Y	ME/GE =	−0.199_(0.077)_+1.21_(0.112)_OMD	0.877	0.037
3c	Z	ME/GE =	−0.079_(0.042)_+1.128_(0.067)_DOMD	0.862	0.046
3d	AA	ME/GE =	0.028_(0.019)_+0.918_(0.029)_GED	0.917	0.032
3e	AB	ME/GE =	−0.368_(0.228)_+0.31_(0.137)_DMD+1.566_(0.328)_N+0.037_(0.328)_GE	0.897	0.042
3f	AC	ME/GE =	−0.656_(0.198)_+0.66_(0.136)_OMD+1.022_(0.262)_N−0.325_(0.782)_EE+0.042_(0.011)_GE	0.938	0.034
3g	AD	ME/GE =	−0.486_(0.144)_+0.646_(0.091)_DOMD+1.014_(0.203)_N+0.035_(0.009)_GE	0.924	0.038
3h	AE	ME/GE =	0.093_(0.025)_+0.757_(0.053)_GED+0.542_(0.157)_N	0.946	0.043
4a	AF	DE/GE =	0.023_(0.054)_+1.015_(0.082)_DMD	0.817	0.034
4b	AG	DE/GE =	−0.181_(0.063)_+1.238_(0.092)_OMD	0.823	0.023
4c	AH	DE/GE =	−0.077_(0.053)_+1.18_(0.084)_DOMD	0.812	0.035
4d	AI	DE/GE =	0.095_(0.047)_+1.076_(0.073)_GED	0.821	0.024
4e	AJ	DE/GE =	0.454_(0.089)_+0.444_(0.125)_DMD−0.398_(0.072)_NDF+5.165_(1.198)_EE	0.879	0.037
4f	AK	DE/GE =	−0.838_(0.21)_+0.731_(0.145)_OMD+0.871_(0.312)_N+0.052_(0.012)_GE	0.907	0.022
4g	AL	DE/GE =	0.082_(0.06)_+0.644_(0.132)_DOMD+0.955_(0.288)_N+2.955_(0.765)_EE	0.912	0.024
4h	AM	DE/GE =	0.089_(0.044)_+0.587_(0.176)_GED+0.902_(0.235)_N+2.978_(0.565)_EE	0.915	0.021

DE, digestible energy content (MJ/kg DM); ME, metabolizable energy content (MJ/kg DM); GE, gross energy content (MJ/kg DM); DMD, DM digestibility (kg/kg), OMD, OM digestibility (kg/kg), DOMD, digestible OM in DM (kg/kg); GED, GE digestibility (MJ/MJ); ADF, acid detergent fiber (kg/kg DM); NDF, neutral detergent fiber (kg/kg DM); N, nitrogen (kg/kg DM); EE, diethyl ether extract (kg/kg DM); tdCP, total digestible CP content (g/100 g DM); tdNDF, total digestible NDF content (g/100 g DM). Data in subscript parentheses are SE values. ^1^ Original equations were developed from the whole data (Table 2, Table 3, Table 4 and Table 5). ^2^ New equations were developed using two-thirds of data. ^3^ Mean prediction error.

**Table 7:** Internal validation of equations developed from two-thirds of the whole data (*n* = 44) using the remaining one-third of data (*n* = 2).

Equation	Parameters Predicted	Predicted Data	Actual Data	MPE ^1^	SE ^2^	R^2^	R_c_^3^		P^4^–A^5^		
								Mean	SD ^6^	Min ^7^	Max ^8^
A	DE	11.71	11.53	0.010	0.003	0.91	0.89	0.179	0.645	−1.142	1.546
B	DE	11.54	11.53	0.015	0.002	0.83	0.91	0.008	0.876	−1.948	1.553
C	DE	11.46	11.53	0.014	0.003	0.79	0.87	−0.074	0.976	−2.638	1.446
D	DE	11.53	11.53	0.011	0.003	0.91	0.92	0.180	0.741	−1.045	2.063
E	DE	11.67	11.53	0.012	0.003	0.90	0.78	0.136	0.772	−0.812	2.128
F	DE	11.46	11.53	0.011	0.002	0.92	0.83	−0.073	0.643	−1.032	1.194
G	DE	11.53	11.53	0.015	0.002	0.90	0.88	−0.299	0.737	−1.429	1.108
H	DE	11.49	11.53	0.013	0.002	0.89	0.84	−0.045	0.782	−1.747	1.571
I	ME	10.14	9.89	0.022	0.008	0.90	0.79	0.251	0.745	−0.823	1.930
J	ME	9.97	9.89	0.027	0.008	0.84	0.91	0.085	0.998	−1.810	1.851
K	ME	10.06	9.89	0.025	0.009	0.79	0.87	0.168	0.862	−1.467	2.161
L	ME	9.59	9.89	0.012	0.002	0.84	0.71	−0.301	0.681	−2.123	0.657
M	ME	9.91	9.89	0.053	0.012	0.86	0.75	0.023	0.854	−0.661	2.371
N	ME	10.70	9.89	0.036	0.012	0.84	0.69	0.807	0.559	−0.075	2.048
O	ME	9.02	9.89	0.053	0.005	0.83	0.65	−0.872	0.694	−1.935	0.490
P	ME	9.98	9.89	0.040	0.005	0.90	0.86	0.095	0.425	0.149	1.646
Q	ME	9.96	9.89	0.020	0.009	0.95	0.77	0.068	0.716	−0.870	2.081
R	ME	8.96	9.89	0.019	0.009	0.95	0.68	0.067	0.730	−0.831	2.048
S	ME	9.95	9.89	0.018	0.009	0.95	0.74	0.060	0.680	−0.682	1.777
T U	ME ME	9.95	9.89	0.019	0.010	0.93	0.81	0.060	0.803	−1.121	2.228
		9.94	9.89	0.018	0.009	0.95	0.74	0.055	0.674	−0.843	1.754
V	ME	10.12	9.89	0.067	0.008	0.87	0.71	0.124	0.662	−0.826	1.504
W	ME	9.81	9.89	0.071	0.008	0.85	0.69	−0.080	0.676	−0.906	1.151
X	ME/GE	0.62	0.62	0.018	0.006	0.71	0.90	−0.006	0.063	−0.072	0.195
Y	ME/GE	0.62	0.62	0.012	0.005	0.86	0.91	0.008	0.044	−0.047	0.148
Z	ME/GE	0.62	0.62	0.061	0.004	0.91	0.89	0.006	0.036	−0.083	0.110
AA	ME/GE	0.62	0.62	0.014	0.005	0.74	0.78	−0.001	0.062	−0.159	0.172
AB	ME/GE	0.62	0.62	0.016	0.006	0.79	0.92	0.001	0.057	−0.096	0.187
AC	ME/GE	0.62	0.62	0.012	0.004	0.86	0.93	−0.005	0.047	−0.065	0.153
AD	ME/GE	0.62	0.62	0.012	0.004	0.88	0.94	0.003	0.043	−0.045	0.145
AE	ME/GE	0.62	0.62	0.013	0.004	0.78	0.91	0.002	0.056	−0.126	0.178
AF	DE/GE	0.64	0.64	0.024	0.005	0.92	0.73	−0.010	0.032	−0.061	0.040
AG	DE/GE	0.64	0.64	0.015	0.003	0.94	0.69	0.001	0.027	−0.030	0.088
AH	DE/GE	0.64	0.64	0.014	0.005	0.88	0.89	−0.004	0.040	−0.068	0.097
AI	DE/GE	0.64	0.64	0.013	0.004	0.93	0.88	−0.003	0.041	−0.064	0.087
AJ	DE/GE	0.64	0.64	0.013	0.004	0.95	0.75	0.002	0.031	−0.063	0.060
AK	DE/GE	0.64	0.64	0.016	0.005	0.91	0.69	−0.004	0.040	−0.049	0.115
AL	DE/GE	0.64	0.64	0.011	0.004	0.95	0.79	−0.004	0.032	−0.078	0.058
AM	DE/GE	0.64	0.64	0.012	0.003	0.95	0.87	−0.005	0.040	−0.062	0.091

DE, digestible energy content (MJ/kg DM); ME, metabolizable energy content (MJ/kg DM); GE, gross energy content (MJ/kg DM). ^1^ Mean prediction error. ^2^ Standard error for predicted data and actual data. ^3^ Lin’s concordance correlation coefficient. ^4^ Predicted data. ^5^ Actual data. ^6^ Standard deviation. ^7^ Minimum value observed. ^8^ Maximum value observed.

**Table 8:** Validation of equations published elsewhere for prediction of ME concentration using one-third of the present dataset (*n* = 22).

Equation	Reference	Equations Validated	Predicted Data	Actual Data	MPE ^1^	SE ^2^	R^2^	R_c_^3^	P ^4^–A ^5^			
									Mean	SD ^6^	Min ^7^	Max ^8^
AN	[27]	ME = 4.2014+0.0236ADF+0.1794CP	10.14	9.89	0.043	0.367	0.22	0.29	0.168	1.559	−2.80	2.48
AO	[28]	ME = 0.132+0.796DE	9.31	9.89	0.042	0.156	0.90	0.31	−0.661	0.661	−1.90	0.69
AP	[29]	ME = 4.184×1.01×(DE/4.184−0.45)	9.74	9.89	0.054	0.128	0.90	0.43	−0.227	0.545	−0.93	1.10
AQ	[19]	ME = 16×DOMD	10.00	9.89	0.062	0.259	0.62	0.27	0.032	1.098	−2.54	2.10
AR	[16]	ME = 0.37+14.2DOMD + 7.7CP	9.85	9.89	0.083	0.242	0.64	0.37	−0.125	1.028	−2.42	2.15
AS	[16]	ME = 9.1+15.3 CP	10.29	9.89	0.053	0.322	0.55	0.59	0.317	1.364	−2.27	2.31
AT	[16]	ME = 18.9+13.3 NDF	10.22	9.89	0.051	0.428	0.11	0.48	0.247	1.814	−3.55	3.14
AU	[15]	ME = 0.815×DE	9.40	9.89	0.052	0.148	0.90	0.39	−0.574	0.626	−1.77	0.78
AV	[15]	ME = −0.232+13.9DOMD+10.05CP	9.24	9.89	0.072	0.241	0.64	0.57	−0.732	1.021	−2.93	1.61
AW	[3]	ME = 15.0−38.9N+34.7EE−10.1ADF−8.07Ash	9.37	9.89	0.054	0.556	0.30	0.62	−0.606	2.357	−4.83	3.16
AX	[3]	ME = −2.238+18.52 DOMD	9.34	9.89	0.082	0.248	0.62	0.42	−0.630	1.050	−3.10	1.54
AY	[3]	ME = 0.432+15.44DMD	9.78	9.89	0.062	0.186	0.81	0.67	−0.192	0.789	−1.24	1.52
AZ	[3]	ME = 1.464+0.723DE	9.80	9.89	0.051	0.168	0.90	0.64	−0.171	0.714	−1.60	1.19

DE, digestible energy content (MJ/kg); ME, metabolizable energy content; ADF, acid detergent fiber (g/kg DM); NDF, neutral detergent fiber (g/kg DM); N, nitrogen (g/kg DM); EE, diethyl ether extract (g/kg DM); CP, crude protein (g/kg DM); DMD, DM digestibility (kg/kg); DOMD, digestible OM in DM (kg/kg). ^1^ Mean prediction error. ^2^ Standard error for predicted data and actual data. ^3^ Lin’s concordance correlation coefficient. ^4^ Predicted data. ^5^ Actual data. ^6^ Standard deviation. ^7^ Minimum value observed. ^8^ Maximum value observed.
